# Late-onset capsular bag distention syndrome resistant to laser treatment: a case report

**DOI:** 10.1093/jscr/rjab278

**Published:** 2021-07-08

**Authors:** Khalid Saad Almousa, Mohammed Nabeel Refka

**Affiliations:** Division of Ophthalmology, King Abdulaziz Medical City, Riyadh, Saudi Arabia; Division of Ophthalmology, King Abdulaziz Medical City, Riyadh, Saudi Arabia

**Keywords:** case report, capsular, bag, distention, late, cataract

## Abstract

Capsular bag distention syndrome (CBDS) or capsular block syndrome is a rare complication of cataract surgery. Neodymium-doped yttrium aluminum garnet (Nd:YAG) laser usually is effective treatment for CBDS. Rarely, surgical intervention is required in resistant cases (as in our case). Herein, we present the case of a 58-year-old male who presented to us with this condition.

## INTRODUCTION

Capsular bag distention syndrome (CBDS) or capsular block syndrome is characterized by the entrapment of fluid between the intraocular lens (IOL) and the posterior capsule. The estimated incidence of CBDS is 0.73% [1]. It has been classified based on the onset time by Miyake *et al*. [2] as intra-operative (after hydrodissection), early post-operative (1–15 days after the surgery) and late post-operative (lacteocrumenasia).

Clinically, the patients can be asymptomatic or present with decreased visual acuity in the affected eye because of the entrapped milky fluid and/or myopic shift [3]. The preferred treatment option is Neodymium-doped yttrium aluminum garnet (Nd:YAG) laser posterior capsulotomy for the majority of symptomatic CBDS cases. However, in some cases (as in our case), surgical intervention is needed to evacuate the turbid fluid, either by anterior or posterior approaches.

## CASE DESCRIPTION

A 58-year-old male with a history of diabetes mellitus and hypertension presented at our clinic complaining of gradual painless decreased vision in his left eye over the last year. He underwent cataract surgery for both eyes outside our facility 2 years before the presentation.

There was no history of pain, photophobia or floaters. His best corrected visual acuity during the first visit was 20/28 in the right eye and 20/2000 (ability to count fingers at 4 feet) in the left eye. Intra-ocular pressure was 18 mmHg in the right eye and 20 mmHg in the left eye. Subjective refraction did not yield better vision in the left (affected) eye.

**Figure 1:**
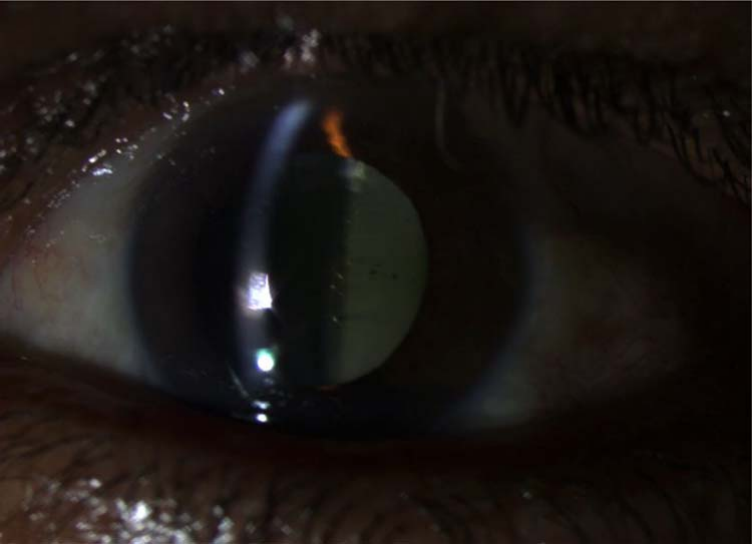
Pre-operative slit lamp photo showed turbid fluid behind the IOL, and the IOL pitting indicates a previous trial of YAG capsulotomy.

On examination, both eyes showed a quiet conjunctiva with clear corneas and quiet pseudophakia. Anterior chamber was deep and equal in both eyes. Left eye showed dense turbid fluid behind the IOL, with multiple pitting on the IOL, indicating a previous trial of Nd:YAG capsulotomy (Fig. 1). Posterior capsule could not be visualized by the slit lamp. Right eye showed a clear capsular bag with a good view of the fundus. Fundus exam showed mild non-proliferative diabetic retinopathy and dry macula in the right eye and no view of the fundus in the left eye.

**Figure 2:**
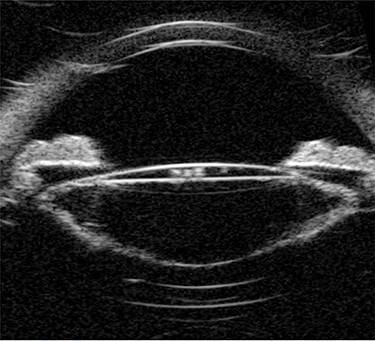
Pre-operative UBM shows a markedly distended capsular bag and thickened posterior capsule.

Ultrasound biomicroscopy (UBM) showed dense turbid fluid behind the IOL with varied echogenicity, a severely distended capsular bag, and a thick irregular posterior capsule (Fig. 2). Based on the previous findings, a diagnosis of late onset CBDS was made.

Due to the poor visibility of the posterior capsule and the overblown capsular bag, surgical intervention using a posterior approach was selected to facilitate surgical capsulotomy without disturbing the position of the IOL and to have better access to aspirate the trapped fluid.

**Figure 3:**
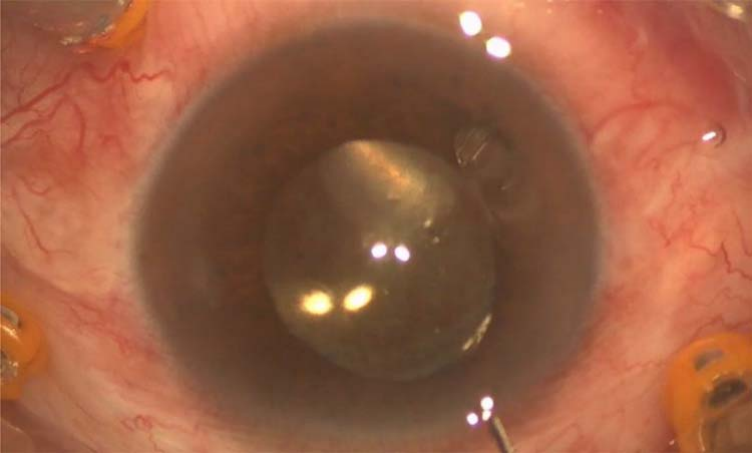
Intra-operative photo showed egression of turbid fluid to the anterior chamber.

**Figure 4:**
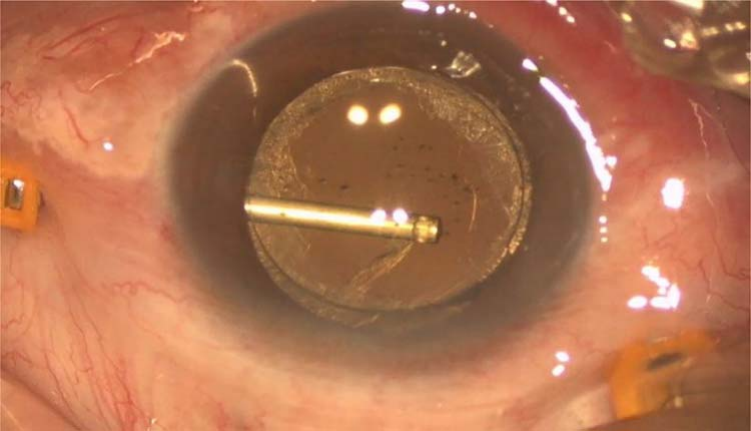
Intra-operative photo showed the posterior capsulotomy using 23G vitrector.

Pars plana vitrectomy and posterior capsulotomy were done. After placing three 23-gauge trocars, a corneal paracentesis was made. After IOL tapping, the entrapped turbid fluid escaped to the anterior chamber (Fig. 3). Anterior vitrectomy and posterior capsulotomy were done using a 23-gauge vitrector (Fig. 4).

During the first post-operative visit, the patient exhibited better vision with a visual acuity of 20/125. The patient reached 20/60 vision after 1 month post-operatively.

## DISCUSSION

Several risk factors for CBDS have been reported in the literature. Residual cortical material is a well-known risk factor, resulting in a milky fluid due to the secretion of alpha crystalline proteins [4]. Other factors include retainment of the ophthalmic viscoelastic device possibly by an osmotic gradient mechanism [2, 3, 5], longer axial length [1] and smaller continuous curvilinear capsulorhexis or larger IOL design because of which the IOL and anterior capsule overlap area is larger, resulting in, the occlusion of the intra-capsular fluid egression pathway [6].

Various diagnostic tools have been used to diagnose CBDS, including slit-lamp biomicroscopy, UBM, anterior segment optical coherence tomography and Scheimpflug-based photography [7]. *Propionibacterium acnes* (*P. acnes*) has been reported in some studies, following the diagnostic aspiration of intracapsular fluid for CSBD [8, 9]. Thus, *P. acnes* might play a role in the pathophysiology of the disease, keeping in mind that *P. acnes* endophthalmitis after Nd:YAG capsulotomy has been reported [10, 11]. Therefore, careful assessment to rule out chronic endophthalmitis is important since the Nd:YAG capsulotomy might induce or worsen endophthalmitis.

Pars plana vitrectomy and posterior capsulotomy for CBDS have not been adequately studied. As in our case, surgical intervention by pars plana vitrectomy and posterior capsulotomy has several advantages over other approaches, including a lower risk of *P. acne* endophthalmitis or inflammation, which might be aggravated by Nd:YAG or anterior approach capsulotomy [4], and the prevention of a possible rise in the intraocular pressure caused by collagen in the milky fluid that might clog the trabecular meshwork [12].

However, apart from the known possible complications of pars plana vitrectomy, it is also considered to be more invasive and costly [4].

Bhattacharjee *et al*. [13] reported 11 cases with delayed onset CBDS treated surgically, and all the patients ended up with 20/20 vision, indicating a good prognosis for this syndrome after proper management.

## CONFLICT OF INTEREST STATEMENT

None declared.

## References

[1] Kim HK, Shin JP. Capsular block syndrome after cataract surgery: clinical analysis and classification. J Cataract Refract Surg 2008;34:357–63. 18299057 10.1016/j.jcrs.2007.11.026

[2] Miyake K, Ota I, Ichihashi S, Miyake S, Tanaka Y, Terasaki H. New classification of capsular block syndrome. J Cataract Refract Surg 1998;24:1230–4. 9768398 10.1016/s0886-3350(98)80017-5

[3] Vélez M, Velásquez LF, Rojas S, Montoya L, Zuluaga K, Balparda K. Capsular block syndrome: a case report and literature review. Clin Ophthalmol 2014;8:1507–13. 25152612 10.2147/OPTH.S67407 PMC4140233

[4] Galvin JC, Berdoukas P, Fung AT. Two cases of very late-onset capsular bag distension syndrome. Am J Ophthalmol Case Rep 2018;10:268–70. 29780949 10.1016/j.ajoc.2018.03.019 PMC5956720

[5] Mastropasqua L, Toto L, De Nicola G, Nubile M, Carpineto P. OCT imaging of capsular block syndrome with crystalline cortical remnants in the capsular bag. Ophthalmic Surg Lasers Imaging 2009;40:399–402. 19634745 10.3928/15428877-20096030-08

[6] Wang JC, Cruz J. Late postoperative capsular block syndrome: entrapment of liquefied after- cataract by capsular bend. J Cataract Refract Surg 2005;31:630–2. 15811757 10.1016/j.jcrs.2004.06.054

[7] Zhu XJ, Zhang KK, Yang J, Ye HF, Lu Y. Scheimpflug imaging of ultra-late postoperative capsular block syndrome. Eye 2014;28:900–4. 24833180 10.1038/eye.2014.107 PMC4094802

[8] Dhaliwal DK, Farhi P, Eller AW, Kowalski RP. Late capsular block syndrome associated with Propionibacterium acnes. Arch Ophthalmol 2011;129:246–7. 21320976 10.1001/archophthalmol.2010.356

[9] Kollias AN, Vogel MA, de Kaspar HM, Lackerbauer CA, Grueterich M. Propionibacterium acnes in capsular bag distension syndrome. J Cataract Refract Surg 2010;36:167–9. 20117721 10.1016/j.jcrs.2009.06.047

[10] Carlson AN, Koch DD. Endophthalmitis following Nd:YAG laser posterior capsulotomy. Ophthalmic Surg 1988;19:168–70. 3258419

[11] Chaudhry M, Baisakhiya S, Bhatia MS. A rare complication of Nd-YAG capsulotomy: propionibacterium acnes endopthalmitis. Nepal J Ophthalmol 2011;3:80–2. 21505550 10.3126/nepjoph.v3i1.4283

[12] Qu J, Bao Y, Li M, Zhao M, Li X. Surgical management of late capsular block syndrome. J Cataract Refract Surg 2010;36:1687–91. 20870114 10.1016/j.jcrs.2010.02.026

[13] Bhattacharjee H, Bhattacharjee K, Bhattacharjee P, Das D, Gogoi K, Arati D. Liquefied after cataract and its surgical treatment. Indian J Ophthalmol 2014;62:580–4. 24881605 10.4103/0301-4738.129771 PMC4065509

